# Analysis of centre of pressure trajectories and plantar pressure distribution to map development of foot-ground interactions from new to confident walking infants

**DOI:** 10.1371/journal.pone.0321632

**Published:** 2025-04-16

**Authors:** Eleonora Montagnani, Stewart C. Morrison, Carina Price

**Affiliations:** 1 Unit for Visually Impaired People, Italian Institute of Technology, Genoa, Italy; 2 School of Life Course and Population Sciences, Faculty of Life Sciences and Medicine, King’s College London, London, United Kingdom; 3 Center for Human Movement and Rehabilitation Research, University of Salford, Salford, United Kingdom; University College Dublin, IRELAND

## Abstract

Independent walking is a crucial milestone, allowing infants to explore their environment efficiently. This phase introduces complex foot-ground interactions. However, previous studies have focused on either center of pressure (CoP) or plantar pressure distribution alone, often losing critical information. The impact of variables like body weight, height, and foot size on pressure distribution in infants remains also underexplored. Our study uses continuous statistical approaches to comprehensively analyze anterior-posterior (AP) and medio-lateral (ML) trajectories of CoP and plantar pressure distribution in infancy, mapping foot-ground interactions from new to confident walking stages. Thirty-nine infants walked across an EMED xl platform as new and then confident walkers. Frames of pressure steps were exported and processed in Matlab 2019b. Upon data normality assessment, AP and ML trajectories of new and confident walking steps were compared with parametric two-sample paired SPM1d t-test. Plantar pressure distribution between new and confident walking were compared using the nonparametric two-sample paired SPM1d t-test. Nonparametric linear regression analysis at pixel-level considered variables like body weight, height, foot dimensions, and walking experience, ensuring only non-correlated items were included. Our analyses revealed significant changes in CoP and plantar pressure distribution from new to confident walkers. New walkers initially contact the ground with the central part of their foot, while confident walkers show a more posterior initial contact. Confident walkers also exhibit more medial heel contact and a progression of CoP trajectories closer to the foot’s longitudinal axis. Regression analysis indicated that increasing walking experience significantly predicts higher pressure in the lateral and central forefoot. These findings underscore the importance of combining multi-segment joint analysis with plantar pressure data to fully understand foot development during infancy. This project highlighted key aspects of the unique biomechanics of infants’ foot development, emphasizing the need for further research to enhance understanding and inform clinical practices.

## Introduction

Independent walking marks the developmental period when the foot is the primary surface in contact with the external environment, allowing infants to move unsupported and explore their surrounding space. Due to the emergence of independent walking skills, different loading demands are placed upon the feet [1], resulting in the development of new and complex foot-ground interactions. Previous studies have analysed either centre of pressure (CoP) [2–5] or plantar pressure distribution [6–9] in infancy to investigate the development of foot function.

Few studies have explored plantar pressure and CoP together, and there are limitations with how these data have been treated statistically [2,9]. Both CoP and plantar pressure distribution data have been analysed using discrete statistical approaches. With these, the information encoded in the 1 and 2-dimensions of the data is simplified into single values. As an example, Hallemans, De Clercq [2] averaged all the point application in both the AP and ML trajectories of the CoP, losing information related to their changes across the stance phase. Similarly, studies analysing plantar pressure distribution in infancy reduced the data spatial resolution using masking approaches to divide the foot in region of interests [9]. As a result, single pressure values are analysed for each region, losing information as to where pressure changes specifically occur on the plantar surface of the feet.

As well as limitations relating to statistical analysis, there is no exploration of the influence of variables such as body weight and height foot size to changes in pressure distribution in infancy, but studies in older children suggest their importance. Accordingly, it has been found that in 4–7 year old children, body weight accounted for 20% and 14% of the peak plantar pressure in the 3^rd^ metatarsal head and medial midfoot respectively [10]. Unger and Rosenbaum [11] normalised force data to body weight in infants to explore the longitudinal development of pressure related to gait changes as opposed to anthropometry at 3-month windows. Concurrent with increases in body weight in infancy, this phase of development is characterised by rapid changes in body composition, size and proportions [12]. The foot grows approximately 24 mm per year for the first three years of life [13], increasing the contact area with the floor available to distribute force [4]. Irrespective of any changes to gait biomechanics influencing pressure distribution and the trajectory of the CoP, such modifications could impact upon plantar pressure variables as infants become confident walkers.

Therefore, we aimed to use continuous statistical approaches for analysis of CoP and plantar pressure distribution, to comprehensively map the complex development of foot-ground interactions in infants from new to confident walking stages. To achieve this, we will: 1) quantify characteristics and differences of CoP trajectories and plantar pressure distribution when walkers are new then confident, and 2) predict changes in plantar pressure distribution using developmentally related variables.

## Methods

### Ethical approval

This study originated from a two-site longitudinal research study as part of the Great Foundation project [14]. Ethical approval was obtained from the ethics committees of the Schools of Health Sciences at the University of Brighton (LHPSCREC 17–11) and the University of Salford (HSCR161779). Written informed consent was approved by the ethical committees and it was obtained from parents/legal guardians of the participants.

### Participants

Participants were recruited from June 2017 to March 2020 to participate within the longitudinal study. Thirty-nine infants (20 females) were recruited in the Northwest (University of Salford) and Southeast (University of Brighton) of England. Infants were included in the study if they were born within 37–42 weeks of pregnancy, had no signs or history of musculoskeletal and/or neurological disorders, audio, visual or sensory impairment, and were born above the fourth percentile for weight. They were excluded if they had family history or have been referred for consultation of suspected musculoskeletal or neurological condition, or if they were taking medicines (which was used as an indicator of health issues) [14].

### Data collection

Data was collected at the Human Movement Laboratories of both Universities. Infants were invited to attend data collection on two occasions, defining two stages of walking development:

1. New walking: infants taking their first 3–5 consecutive steps, independently.
2. Confident walking: infants taking 10–15 steps independently, being able to navigate around objects, interact with parents and carry small toys while walking.

Parents were asked to identify these stages and report to the lead researcher, using videos or images as confirmation of stage attainment. Following achievement of the required stage, infants had 21 days to attend the data collection sessions or were excluded from the study. Informed consent was given from parents at each data collection session on behalf of their infant. Descriptive information of the participants is given in Table 1.

**Table 1:** Participant descriptive information at the two stages of walking development.

	New walking				Confident walking			
Measures	Min	Mean	SD	Max	Min	Mean	SD	Max
**Age at visit (months)**	9.1	13.3	1.6	16.6	12.3	15.6	1.8	20.2
**Age at milestone (months)**	8.6	12.8	1.5	16.2	11.8	15.1	1.8	19.6
**Days since milestone (days)**	7.0	14.5	5.1	21.0	3.0	15.2	5.5	21.0
**Mass (kg)**	8.0	10.3	1.3	13.8	8.5	11.0	1.3	14.0
**Height (cm)**	68.5	74.7	3.1	81.8	71.9	78.4	3.5	85.0
**Foot length (cm)**	9.7	11.4	0.7	12.6	10.5	11.9	0.8	13.6
**Absolute foot width (cm)**	4.4	5.1	0.4	6.0	4.3	5.2	0.4	6.2
**Normalised foot width (foot width/ foot length)**	39.8	45.6	3.5	55.14	36.1	45.7	9.7	80

### Testing procedure

At each stage of walking, plantar pressure data was collected using a Novel EMED xl platform (100 Hz, 4 sensors per cm^2^; Munich, Germany). The platform was placed in the middle of a nursery-style environment, where infants walked freely, at self-selected speed and self-directed (i.e., infants were not asked to walk in straight lines from one point of the testing space to another). The pressure system was triggered for 60 seconds once infants started walking, but the trials were ended before the full period if infants ceased walking. We tried capturing data for at least 10 minutes, with each trial lasting for a minute. For this study, we selected three trials for each participant. Walking was recorded with HD videos (University of Brighton: Vicon Bonita 720c; Oxford, U.K/ University of Salford: Logitech HD Pro Webcam), which were used to identify the walking tasks performed (e.g., walking in straight lines, squatting, walking while turning), and pressure steps were coded accordingly [6].

### Data pre-processing

For each infant and walking stage, pressure steps that were taken in a straight line were saved individually using the standard EMED software (Novel, Munich, Germany). Steps were processed only if they were taken within the platform borders, did not show omission of extensive anatomical parts and included a full step cycle. These were determined through visual inspection. Three left and right steps for each infant at each stage of walking were chosen (n=468). This number was selected to maintain consistency with previous work in the field [4,8,15,16] and reduce computational costs [9]. For CoP analysis, frames of pressure were processed, defined as all the consecutive images composing a maximum pressure picture (MPP), while for plantar pressure distribution, we exported only the respective MPPs. Frames of pressure and MPPs of each infant and walking stage were exported as ASCII text files (Novel Emascii software), and imported into Matlab2019a (The Mathworks Inc, Natick, USA) as 2D numeric matrices for data processing and analysis. Non-zero entries of each frame and MPP corresponded to pixels containing pressure values. Each frame and MPP was positioned in a grid of 36x25 pixels. As spatial orientations of the steps was highly variable due to the testing protocol adopted [1], all frames for each step were vertically rotated using principal component analysis (PCA), to ensure consistent computations of COP trajectories across steps of infant.

### Centre of pressure trajectory data processing

For each step, a 3D structure (36x25xlength of the number of frames in each step) was created and ML and AP trajectories were calculated from the 3D structure using a freely available tool (https://github.com/GallVp/footPress) [17]. New 101-point ML and AP trajectories were obtained using a built-in Matlab function for spline interpolations. Whilst with PCA we consistently oriented the frames of pressure in the Euclidean space, differences were still present as to where those frames were positioned in the matrix (36x25), across both participants and walking stages. Therefore, a common reference system for the respective components of the COP was also created across participants. This procedure was undertaken using the maximum pressure picture (MPP) of each participant. For AP, the most posterior contact point of each step was determined by considering the minimum value (K) present in the y-axis of the reference system of each full image. K was then estimated within (K_WS_) and between infants (K_BS_) to calculate new entries of the within infant AP trajectories at both visits:


$${\text{AP}}_{\text{i}}\left(\text{t}\right)=\text{AP}\left(\text{t}\right)-{\text{K}}_{\text{BS}}\;(\text{cm})$$

For ML, we found the longitudinal axis of each full pressure image passing through its centroid (J) using PCA, as reported by Montagnani et al. (2021). A within-participant centroid (J_WS_) was estimated across the full images at each visit, and we calculated the between-infants centroid (J_BS_) across visits. Such value was then used to calculate new entries of the within-participant ML trajectories:


$${\text{ML}}_{\text{i}}\left(\text{t}\right)=\text{ML}\left(\text{t}\right)-{\text{J}}_{\text{BS }}\;\left(\text{cm}\right)$$

Point applications of the within-infants ML trajectories with negative and positive values (falling on the left and right of the longitudinal axis of the images) were considered as lateral and medial point COP applications, respectively.

Finally, both ML and AP trajectories of each infant were normalised to the respective length and width of the feet, to account for differences between the infants’ feet due to growth.

### Maximum peak picture data processing

MPPs were processed according to Montagnani, Morrison [1], and therefore, we will provide a brief description of the framework used. After PCA, we transformed each MPP in point clouds and performed within-infant registrations using an iterative closest point algorithm. Once registered, the coordinates of the point clouds were averaged, resulting in one mean point cloud per participant per foot. We then performed a between-infants registration using a non-rigid coherent point drift algorithm, using an unbiased template chosen to be consistent for all the participants [1].

### Statistical analysis

The SPM1d package (http://www.spm1d.org/) was used for analysis of CoP and plantar pressure distribution. The normalised AP and ML trajectories were checked for normality with SPM1d normality test for 1D data. Normality tests failed to reach significance at α=0.05, suggesting insufficient evidence against the null hypothesis of normality. Thus, a parametric two-sample paired SPM1d t-test was used to compare AP and ML trajectories of infants when they are new and confident walkers (α=0.05). As the SPM1d package does not yet provide with a normality test to check normality of 2D data, plantar pressure distribution between new and confident walking were compared using the nonparametric two-sample paired SPM1d t-test (α=0.05). Next, nonparametric linear regression was also performed at pixel level. While we appreciate that using multivariate models yielded higher statistical power, the assumptions of 2D smoothness and topological features estimates of plantar pressure data [18] prevented us to use such analyses.

Prior to linear regression, the assumptions of regression were checked. A correlation matrix was calculated based on Pearson product-moment correlation coefficients (r), to ensure that only items that were not moderate to strongly correlated with each other (r ≥0.5) were considered as independent variables within the broader analysis. This calculation was undertaken as indicators of body maturation such as height, weight and feet dimensions are likely related to each other as they increase as part of the overall growth. This is also why the normalised foot width (as the ratio between foot width and length, divided by 100) has been added in the regression model, to account for percent of growth of foot width with respect to foot length. Other independent variables considered initially in the regression model were body weight, height, foot length, foot width and walking experience (expressed as the total days infants took to be defined as new to walking and walking confidently).

## Results

### Characteristics and comparison of CoP trajectories from new to confident walking

**Fig 1:**
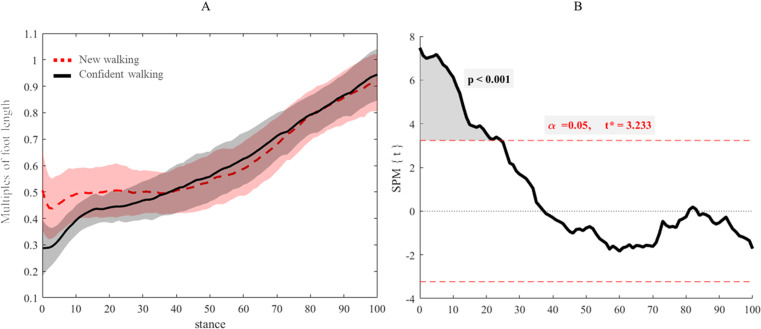
(A) Mean and standard deviation (shades) of normalised anterior-posterior (AP) trajectories to feet lengths of new (red dashed line) and confident walking infants (black line). (B) Parametric SPM1d paired t-test for comparison of the normalised anterior-posterior COP trajectory between walking stages. Critical threshold was exceeded between 0-25% of stance, indicating a significantly increase in posterior loading in confident walkers.

Origins of AP trajectories from the most posterior point of contact were at 50% and 30% of total foot length in new and confident walking, respectively (Fig 1A). Between 0–20% of stance, new walking AP trajectories differed to confident walking (t=3.23, p<0.001) (Fig 1B). From 40% of stance, AP trajectories of new and confident walking almost overlapped (Fig 1A).

**Fig 2:**
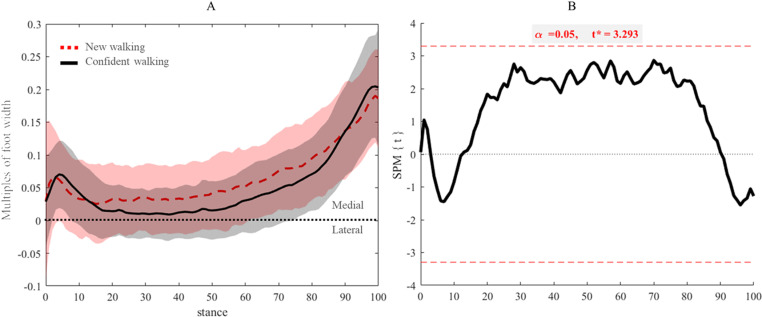
(A) Mean and standard deviation (shades) of normalised medio-lateral (ML) trajectories to feet widths of new (red dashed line) and confident walking infants (black line). Black dashed line represents the longitudinal axis of the foot passing from the second toe to the centre of the heel. (B) Parametric SPM1d paired t-test for comparison of ML components of the COP trajectory between walking stages. Critical threshold was not exceeded across any point in stance, indicating absence of statistical differences.

In both new and confident walking, the ML trajectories showed that CoP was always medial to the foot axis across the entire stance (Fig 2A). In confident walking steps, ML trajectories were shifted more towards the longitudinal axis of the foot from 0–20 and 65–100% of stance during new walking (Fig 2A). However, no significant differences in the progression of the ML trajectories of the CoP were found between the two walking stages (t=3.29, p>0.05) (Fig 2B).

### Comparison of plantar pressure distribution from new to confident walking

**Fig 3:**
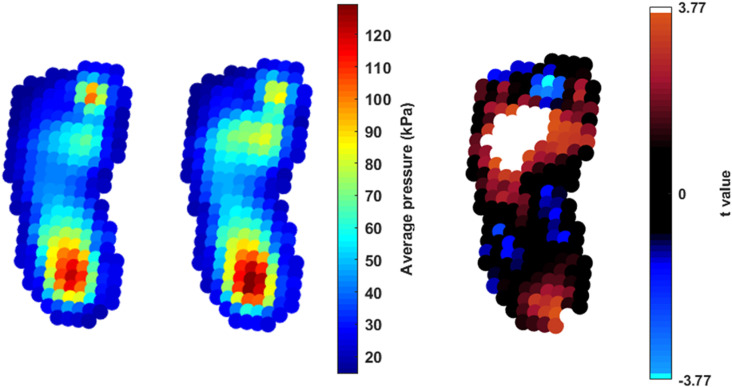
Nonparametric pSPM paired t test for comparison between stages of walking. From left to right: average pressure distribution of new walking, average pressure distribution of confident walking, and raw t value of statistical analysis. The extremes of the colourbar in the inference image (extreme right) reflects t-values needed to reach statistical significance, with alpha set at 0.05. The colourbar for the average pressure distribution images presented different min and max kPa values that were adjusted by the overall max and min values to allow for comparison. Cool and warm colours identify where maximum pressure pictures of confident walking infants had lower and higher peak pressure than new walkers, respectively.

Areas of significant increase in pressure were found in the lateral and central forefoot during confident walking steps (Fig 3C). This was surrounded by a non-significant area of increasing pressure distributed across the forefoot, in the lateral side of the midfoot, and lateral toes (Fig 3C). A point of significant increasing pressure was also detected in the posterior medial border of the heel during confident walking, and an area of non-significant increasing pressure covered the posterior to central heel. In the medial and lateral sides of the midfoot, pressure decreased although not significantly in confident walkers. In the medial toe area (approximately between the hallux and second toe), a point of significantly decreasing pressure was detected in confident walking steps compared to new walking steps, which was surrounded by points of non-significant reduced pressure.

### Correlation matrix and regression analysis

**Fig 4:**
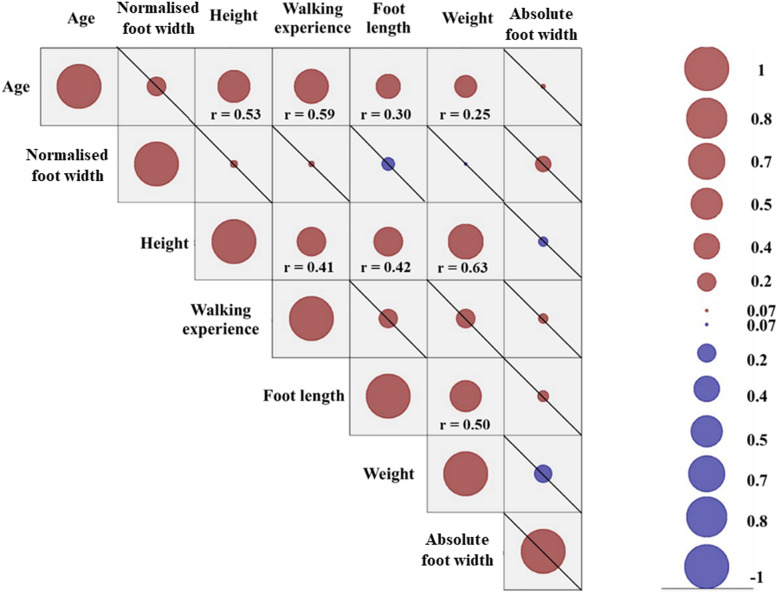
Correlation matrix of the Pearson product-moment correlation coefficient (r) calculated among age, foot proportions, height, walking experience, foot length, weight, and foot width. The size of the circles indicates strength of the correlation, whilst the colour indicates the direction (positive or negative). Results that did not achieved significance were crossed and r were reported only for items that reached significant correlation (p<0.001).

The correlation matrix identified variables that significantly (p<0.001) and moderately correlated (r≥0.5) with each other (Fig 4). Specifically, there was a moderate positive correlation between height and age (r=0.53) and weight (r=0.63). Moderate positive correlations were also detected between body weight and foot length (r=0.50) and walking experience and age (r =0.59) (Fig 4). The independent variables to consider within the regression model were chosen following the considerations reported hereafter.

For example, walking experience moderately and significantly correlated with age only (r=0.59), whilst age also moderately correlated with height (r=0.53). By relying solely on correlation results, age would likely be considered as predictor instead of walking experience, considering that it correlated with more items. However, the age of participants at each visit was not included as it represents only a chronological event and does not explain development [19], as infants attain different stages of motor development at different age [20]. Furthermore, increasing age account for maturation of multiple body systems (musculoskeletal, neurological), and does not represent a specific process of development. Therefore, works in developmental biomechanics using age in predictive model might fail to recognize specific predictors related to foot function changes, leading to generalized consideration.

This work also accounted for changes in normalised foot width (changes in foot width relative to length) within the regression model. This way, it was possible to separate findings related specifically to increasing foot length and width from the influence that the 2D changes in foot size might have had on plantar pressures. It is recognised that other variables related to body maturation, such as tibial length or leg length were not included in the model. In fact, it was decided not to use these variables as they accounted partly for the overall increasing height, and thus the inclusion of height in the initial regression model was considered comprehensive of such changes. Height however was not included in the final regression model as two variables related to maturation were moderately correlated with weight, which was therefore included in the analysis as opposed to height.

**Fig 5:**
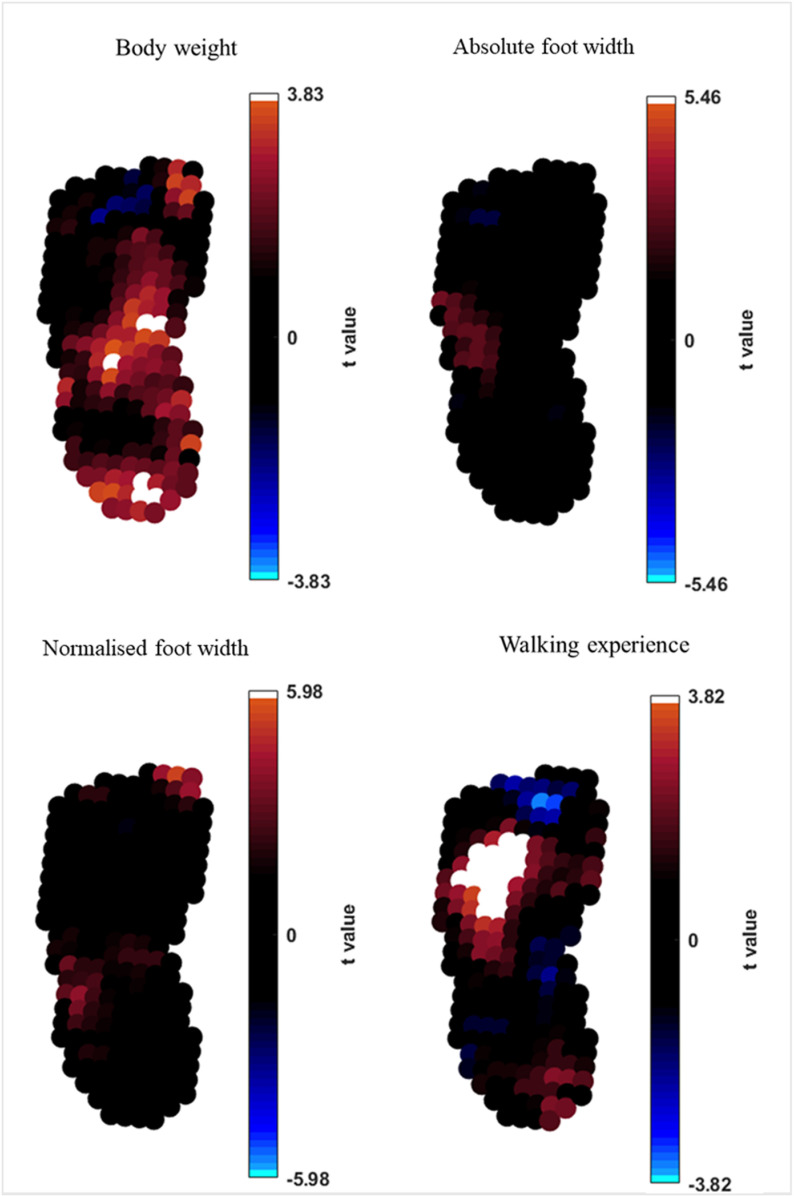
Nonparametric linear regression analysis at pixel level between body weight, foot width, foot proportions, walking experience and maximum pressure pictures. T-values indicates the strength of linear regression between the predictors and peak pressure data. Extremes of the colourbar reflects t-values needed to reach statistical significance, with alpha set at 0.05.

A positive and significant relationship between increasing body weight and pressure was found in the medial midfoot, in the central midfoot and in the posterior border of the heel (p< 0.05; t=+3.83) (Fig 5). The increasing walking experience also demonstrated a significant positive relationship with pressure in the lateral and central forefoot (p<0.05; t=+3.82). Non-significant relationship between walking experience and pressure were found also in the posterior border of the heel (positive relationship) and in the hallux (negative relationship). No relationships were demonstrated between absolute foot width and pressure (p≥0.05; t=±5.46), whilst areas of positive but not significant relationship between normalised foot width and pressure were identified in the lateral midfoot and in the apex of the hallux (p≥0.05; t=±5.98) (Fig 5).

## Discussion

The aim of this work was to map the development of foot-ground interactions as infants became confident walkers, using continuous statistical approaches for analysis of CoP and plantar pressure distribution data.

Our analyses revealed that a series of changes in CoP and plantar pressure distribution took place from new to confident walking. Specifically, we found that, in new walkers, initial foot contact was made using the central part of the foot surface, as the origin of contact found at 50% of the total foot length. Alternatively, steps when walking confidently showed a more posterior initial contact, as the origin of contact found at 30% of the total foot length. Increasing ankle dorsiflexion at initial contact during gait [21] can be identified as the main developmental event behind foot-ground interactions at this stage, leading infants to a more plantigrade initial foot contact. Nevertheless, combination of multi-segment joint analysis with plantar pressure data is required to demonstrate fully such aspects of foot development. Alongside changes in the Anterior Posterior component of the CoP trajectory, the Medio-lateral component of the trajectory highlighted a more accentuated medial contact from 0–20% of stance in steps once walkers were confident. Initial medial heel contact in steps once walkers were confident could explain why pressure in this area significantly increased, as demonstrated by analysis of plantar pressure distributions (Fig 3). We appreciate that only one point of pressure significantly increased from new to confident walking, and thus it would be reasonable to question the pixel resolution of our pSPM approach. With respect to pixel resolution, our previous work reported that the errors due to pixels overlap were below 0.4 mm in both the registration algorithms [1], suggesting a high level of confidence within the present results. With respect to the relevance of this result, one pixel of significant increasing pressure might not be considered determinant to quantify pressure distribution changes. However, the presence of such a restricted area of significance might be caused by the short gap between the two stages of walking. Thus, further studies should be carried out to check whether a longer duration between observations, larger areas of increasing pressure would be present in the heel.

From 20 to 70% of stance, the steps once walking confidently demonstrated a progression of ML trajectories of the CoP closer to the longitudinal axis of the foot compared to new walkers. Although such result is not significant statistically, a less accentuated progression of the CoP trajectories in the medial domain of the foot is a new finding in relation to CoP progression in infancy. Previous works have reported lower stability indices of ML trajectories in steps of confident walking, inferring less ML oscillations of the CoP, hence a more stable gait in confident walkers [2,4]. Although we do not exclude this, our analysis enabled, for the first time, visualisation of ML progression of CoP trajectories over stance, demonstrating the presence of a shift in CoP trajectory rather than oscillations.

The significant increasing pressure in the lateral and central forefoot from new to confident walking could also explain why there is a progression of the CoP closer to the longitudinal axis of the foot in confident walking. In fact, it is plausible to assume that, whilst pressure is reduced medially, it is increased laterally as it starts resembling a more developed contact pattern with more walking experience [22]. This a key aspect in the field of developmental biomechanics, highlighting the importance of combining multi-dimensional plantar pressure data, as there are limited considerations that could be drawn only considering the spatial aspects of pressure changes (i.e., plantar pressure distribution). With regression analysis, we were able to demonstrate that the parameter predicting plantar pressure changes in the lateral and central forefoot was the increasing walking experience, which was positively and significantly associated with changes in pressure in these areas. As a result, it would be possible to surmise that factors associated with increasing walking abilities, such as higher walking speed could be possibly associated with higher pressure in these areas too. This was also reported in previous research with a cohort of infants [23] as well as in older children [24]. Given the ecological setting of our protocol (i.e., infants were free to walk at preferred speed), we did not record walking speed as predicting variable to support such hypothesis. Nevertheless, these results confirm the importance of walking experience as exploratory variable to detect plantar pressure distribution changes during walking.

With more confident walking, the medial component of the CoP trajectory moved closer to the longitudinal axis of the foot than when infants were new to walking. Which could also explain reduction in pressure in the medial midfoot in more confident walking steps. This is an important finding as plantar pressure studies using regions of interest have typically considered the midfoot as a whole area [4,8,15,23], possibly missing information related to its functional independence at early stages of walking development. However, this work confirms that future studies should involve considerations of the medial midfoot as a separate region of interest if discrete analysis approaches are implemented. Important considerations related to the medial midfoot were also made by considering the relationship of body weight with peak pressures in this area as well as in the medial aspect of the foot. Specifically, this is the first time that a significant positive relationship between increasing body weight and peak pressures in the medial midfoot has been reported, and a positive yet not significant relationship between body weight and pressure in the medial aspect of the foot. As a result, body weight variation in infancy underpins changes related to the medial midfoot function. This is possible because the underlying midfoot structures are likely to be susceptible to the increasing weight, as anatomically the medial longitudinal arch supporting the loading is not fully developed yet at these early stages of walking [25].

The characteristics of ML trajectories of the CoP demonstrated that the stance phase at the onset of walking and once walking confidently terminated with an accentuated medial loading, supporting the presence of a terminal stance phase using the medial aspect of the foot. With this, it would have been plausible to expect increasing pressure around the medial toe area from the new to confident walking steps. This, however, significantly reduced, which can be explained by the changes to initial contact pattern. In fact, the shift to an initial rearfoot contact with the floor once walkers were confident could cause a reduction in peak pressure in the forefoot during the terminal stance phase when compared with the midfoot or forefoot contact during steps when new to walking. In fact, pressure would start being distributed just from posterior to anterior areas of the foot as opposed to record a pattern of continuous forward-backward displacement, which might have involved extra medial toe loading throughout the entire stance.

## Limitations

Limitations related to the statistical approach to linear regression data analysis are present in this work. Multivariate analysis exists in the SPM package, but its use is limited to normally distributed data and could not be adopted for 2D pressure analysis due to the underlying SPM assumption of 2D smoothness estimates and topological features estimates of the data. Furthermore, the short duration between the two stages of walking might prevent pressure changes to be predicted by certain maturation variables such as foot width, which did not show changes from the new walking to confident walking. Extending the observation to other stages of walking in later infancy might capture more accentuated changes in foot size, which could have the potential to predict changes in plantar pressures.

## Conclusions

This study presented, for the first time, a comprehensive evaluation of CoP and plantar pressure distribution patterns and changes when infants move from new to confident walking, analysing one of the largest plantar pressure data sets collected in infancy up to date, with high resolution statistical methods. Investigations identified that AP trajectory was shorter in new walking steps and longer in confident walking steps, due to it initiating nearer the heel in the latter walking stage. Alongside this, lateral and central forefoot pressures were higher in the forefoot once walking confidently and the regression analysis identified a significant positive relationship between pressure and walking experience in this area.
